# Localisation inhabituelle du sarcome d'Ewing des parties molles

**DOI:** 10.11604/pamj.2016.23.84.8340

**Published:** 2016-03-11

**Authors:** Youssef Omor, Nabil Moatassim Billah

**Affiliations:** 1 Service d'Imagerie Médicale, Radiologie Centrale, CHU Avicenne, Rabat, Maroc

**Keywords:** Sarcome d′Ewing, parties molles, région cervicale, Ewing's sarcoma, soft parts, cervical region

## Image en medicine

Le sarcome d'Ewing des parties molles est une tumeur mésenchymateuse rare, de mauvais pronostic, qui doit bénéficier d'un diagnostic précoce, afin d'offrir les meilleures chances de survie. Devant l'absence de signes cliniques et radiologiques spécifiques, il semble nécessaire de l'inclure comme diagnostic différentiel devant toute tumeur primitive des parties molles et y penser même devant des localisations inhabituelles. La localisation cervicale est inhabituelle et ne représente que 7% des sarcomes d'Ewing des parties molles. L'imagerie, en particulier l'IRM, permet un bilan lésionnel et d'extensions exhaustives et un suivi thérapeutique. Il s'agit d'un patient âgé de 35 ans, sans antécédent particulier, qui présentait depuis 7 mois une tuméfaction postérieure au niveau de la charnière cervico-dorsale, ayant augmentée progressivement de volume, non douloureuse, qui évoluait dans contexte d'altération de l’état général et d'amaigrissement important. L'examen clinique mettait en évidence une énorme masse ferme, insensible, fixe par rapport au plan superficiel, mesurant environ 174x197x165 mm, non soufflante, sans signes inflammatoires ou circulation veineuse collatérale en regard ni adénopathies locorégionales. Le patient a bénéficié d'une IRM objectivant un volumineux processus lésionnel pariétal de la région cervico-dorsale de signal hétérogène en T2 et T2 FAT SAT (A, B, C). Ce processus tumoral infiltrait le plan musculaire superficiel et profond de la région cervico-dorsale arrivant jusqu'au niveau des muscles para vertébraux à gauche sans signes d'extension rachidienne ni endocanalaire. L'examen anatomapathologique avec étude immuno-histochimique a permis de confirmer le diagnostic de sarcome d'Ewing des parties molles.

**Figure 1:**
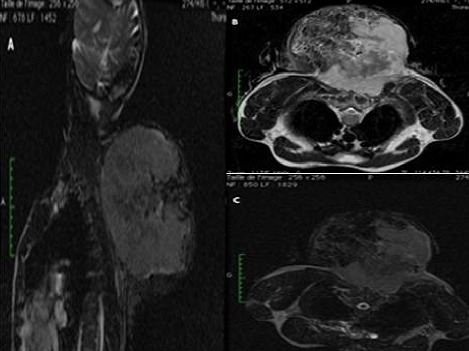
IRM de la région cervico-dorsale en séquences pondérées T2,T2 FAT SAT, et T1 injectée

